# Dietary Protein Affects Gene Expression and Prevents Lipid Accumulation in the Liver in Mice

**DOI:** 10.1371/journal.pone.0047303

**Published:** 2012-10-23

**Authors:** Jessica Schwarz, Daniel Tomé, Annemarie Baars, Guido J. E. J. Hooiveld, Michael Müller

**Affiliations:** 1 Nutrition, Metabolism and Genomics Group, Division of Human Nutrition, Wageningen University, Wageningen, the Netherlands; 2 Netherlands Nutrigenomics Centre, Wageningen, the Netherlands; 3 AgroParisTech, INRA, UMR914 Nutrition Physiology and Ingestive Behavior, Paris, France; Institut Pluridisciplinaire Hubert Curien, France

## Abstract

**Background and Aims:**

High protein (HP) diets are suggested to positively modulate obesity and associated increased prevalence of non-alcoholic fatty liver (NAFLD) disease in humans and rodents. The aim of our study was to detect mechanisms by which a HP diet affects hepatic lipid accumulation.

**Methods:**

To investigate the acute and long term effect of high protein ingestion on hepatic lipid accumulation under both low and high fat (HF) conditions, mice were fed combinations of high (35 energy%) or low (10 energy%) fat and high (50 energy%) or normal (15 energy%) protein diets for 1 or 12 weeks. Effects on body composition, liver fat, VLDL production rate and the hepatic transcriptome were investigated.

**Results:**

Mice fed the HP diets displayed a lower body weight, developed less adiposity and decreased hepatic lipid accumulation, which could be attributed to a combination of several processes. Next to an increased hepatic VLDL production rate, increased energy utilisation due to enhanced protein catabolic processes, such as transamination, TCA cycle and oxidative phosphorylation was found upon high protein ingestion.

**Conclusion:**

Feeding a HP diet prevented the development of NAFLD by enhancing lipid secretion into VLDL particles and a less efficient use of ingested calories.

## Introduction

Within the context of the metabolic syndrome clear links are demonstrated between non-alcoholic fatty liver disease (NAFLD) and insulin resistance, type 2 diabetes mellitus and obesity [1], [2]. NAFLD develops from hepatocellular steatosis via non-alcoholic steatohepatitis (NASH) to fibrosis and can eventually end up in irreversible cirrhosis [2], [3]. In western populations the prevalence of NAFLD is estimated to be between 30–46% [4]. In obese patients the prevalence of developing NASH or fibrosis was found to be even increased to 60–70% [5], [6]. In addition to genetic predisposition, amongst others in apolipoprotein encoding genes [7], [8], environment and lifestyle may affect the development of fatty liver. Patients suffering from NAFLD were observed to consume more food from fast food restaurants and have a more sedentary lifestyle as well as a lower fitness level compared to people not diagnosed with NAFLD [4], [9]. In intervention studies which targeted regression of hepatic lipids, an increase in physical activity was mostly successful in combination with a reduction in energy intake [10], [11].

The amount of hepatic lipids can be modified by the macronutrient composition of the diet by modulating hepatic fatty acid uptake, lipogenesis, fatty acid oxidation and triglyceride secretion [12], [13]. It has been observed that high fat (HF) diets lead to hepatic lipid accumulation in both humans [14], [15] and rodents [16], [17]. Recently, evidence links a high intake of simple carbohydrates and fructose, mainly by consumption of soft drinks, with the development of NAFLD and even NASH [18], [19]. In rats fed a diet rich in fructose, an increase in hepatic fat was likewise observed [20], [21]. In contrast, diets high in protein are suggested to prevent and decrease hepatic lipid content. In rodents, it has been shown that augmenting the amount of protein during high fat feeding results in reduced adiposity and liver fat [22], [23]. In human, the increase in intrahepatocellular lipid (IHCL) deposition induced by a HF diet was reduced when dietary protein was augmented [15]. Most of these studies, however, were short term interventions and long term treatment might lead to different results.

The present study in mice aims at analysing mechanisms reducing hepatic lipid content in response to a HP diet. Mice were fed different combinations of high fat and high protein diets for 1 or 12 weeks to investigate the acute and long term effect of such a diet on body adiposity, liver fat, VLDL production rate and hepatic gene expression. We hypothesise that augmenting the amount of dietary protein affects liver lipid uptake, *de novo* lipogenesis, nutrient oxidation and VLDL secretion.

**Table 1:** Diet Compositions.^1^

	Low fat-normal protein diet (LF-NP)	High fat-normal protein diet (HF-NP)	Low fat-high protein diet (LF-HP)	High fat-high protein diet (HF-HP)
Metabolisable energy, *kJ/g*	16.1	18.5	16.1	18.5
	*g/kg dry matter*			
Total milk protein^2^	140	160	484	580
Cornstarch^3^	361.35	291.3	189.35	80
Sucrose^4^	361.35	291.4	189.35	80
Soybean oil^5^	40	40	40	40
Palm oil^5^	0	120	0	123
AIN 93M mineral mix^6^	35	35	35	35
AIN 93V vitamin mix^6^	10	10	10	10
a-Cellulose^7^	50	50	50	50
Choline^6^	2.3	2.3	2.3	2.3

1 All dietary components were purchased (sources given below) or prepared by UPAE (Unité de Préparation des Aliments Expérimentaux, French National Institute of Agronomic Research, INRA, Jouy en Josas, France).

2 Nutrinov, Rennes, France.

3 Cerestar, Haubourdin, France.

4 Eurosucre, Paris, France.

5 Bailly SA, Aulnay-sous-bois, France.

6 ICN biochemicals, Cleveland, OH.

7 Medias filtrants Durieux, Torcy, France.

## Materials and Methods

### Ethics statement

All animal experiments were approved by the Animal Experimentation Board at Wageningen University (record #2010017) and carried out according to the guidelines of the European Convention of Vertebrate Animals Used for Experimentation, under European Council Directive 86/609/EEC dated November, 1986.

**Figure 1:**
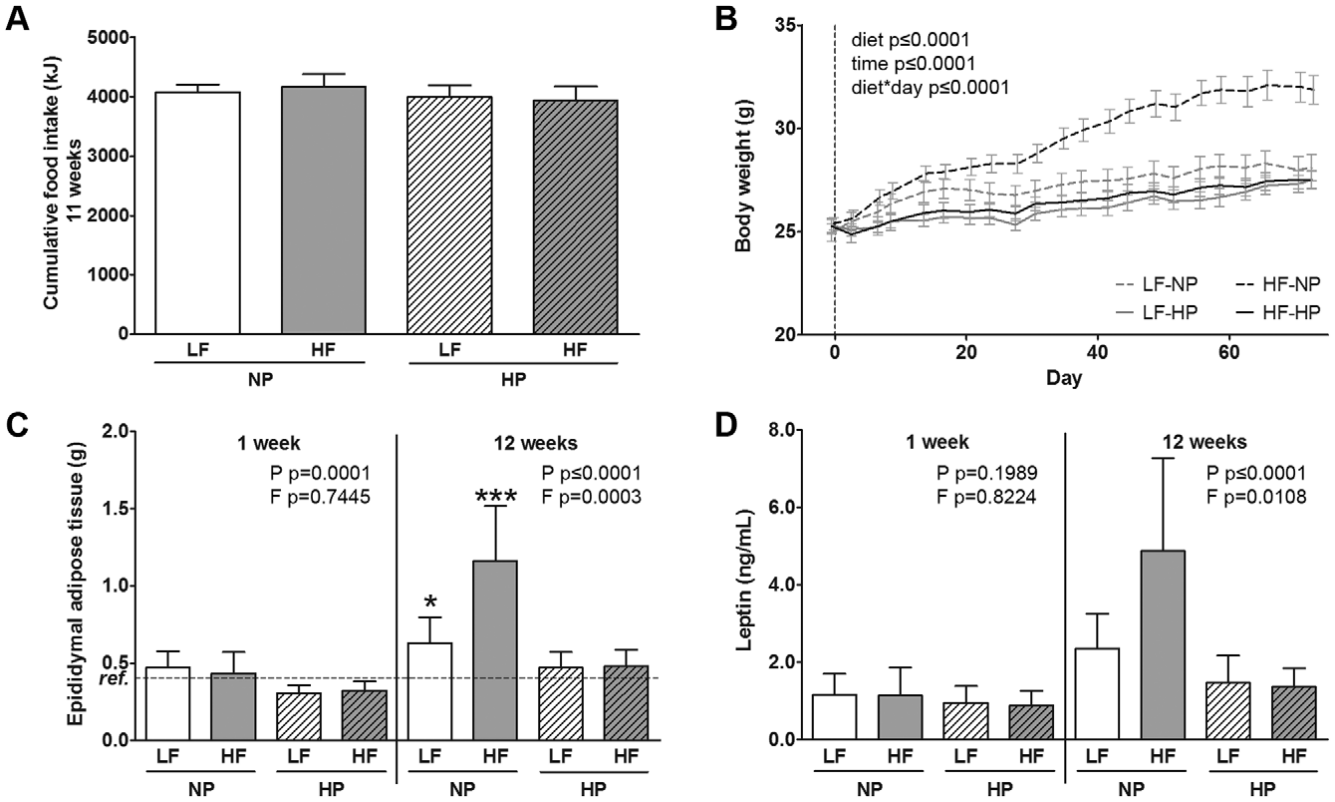
Body composition and food intake. Whereas cumulative food intake (A) did not differ (mean ± SD), *ad libitum* fed C57BL/6J mice (n = 10 per group) in the HF-NP group developed a higher body weight (B) compared to the other groups (mean ± SEM). Epididymal adipose tissue mass (C) and plasma leptin (D) are likewise increased in the HF-NP group. Effects of increased dietary protein (P) and fat (F) within one time point were tested using general linear model with P and F as fixed factors. * represents values significantly different from the reference group (ref.) as determined by one way ANOVA: *p≤0.05, **p≤0.01, ***p≤0.001.

### Animals and diets

Male C57BL/6J mice aged 8 weeks were purchased from Charles River (L'Arbresle, France) and housed in pairs in the light and temperature-controlled animal facility of Wageningen University (12∶12 h reversed light/dark cycle, 20±2°C). At 10 weeks of age, mice were stratified according to their bodyweight into 9 diet groups (n = 10 per group). Diets were provided as powder in specifically designed food cups to minimise spillage. All groups received a low fat-normal protein diet (LF-NP, 10 and 15 energy% from fat and protein, respectively) as run-in of two weeks prior to the dietary intervention. One group was sacrificed after the run-in period to serve as reference group. The other groups either continued on the LF-NP diet or were switched to a high fat-normal protein (HF-NP, 35 and 15 energy% from fat and protein, respectively), a low fat-high protein (LF-HP, 10 and 50 energy% from fat and protein, respectively) or a high fat-high protein (HF-HP, 35 and 50 energy% from fat and protein, respectively) diet which they received for 1 or 12 weeks. For diet compositions see table 1. Body weight was recorded twice a week at the end of the light phase. Food intake was measured daily by weighing back the food cups when replaced with fresh food at the end of the light phase. Spilled feed was collected and taken into account in the analysis. The week prior to sacrifice, mice were habituated to a modified feeding schedule. Food was removed at the beginning of the light phase. One hour after beginning of the dark phase, mice received a calibrated meal of 12.1 kJ to be consumed during 2 hours before getting *ad libitum* access to their habitual diet. The day of sacrifice, mice received the calibrated meal at the habitual time and were anaesthetised with Isoflurane 2 hours later. Blood was taken by orbital puncture and plasma was stored. Liver and epididymal adipose tissue were removed, weighed and snap frozen in liquid nitrogen. Tissue was stored at 80°C until further analysis.

**Figure 2:**
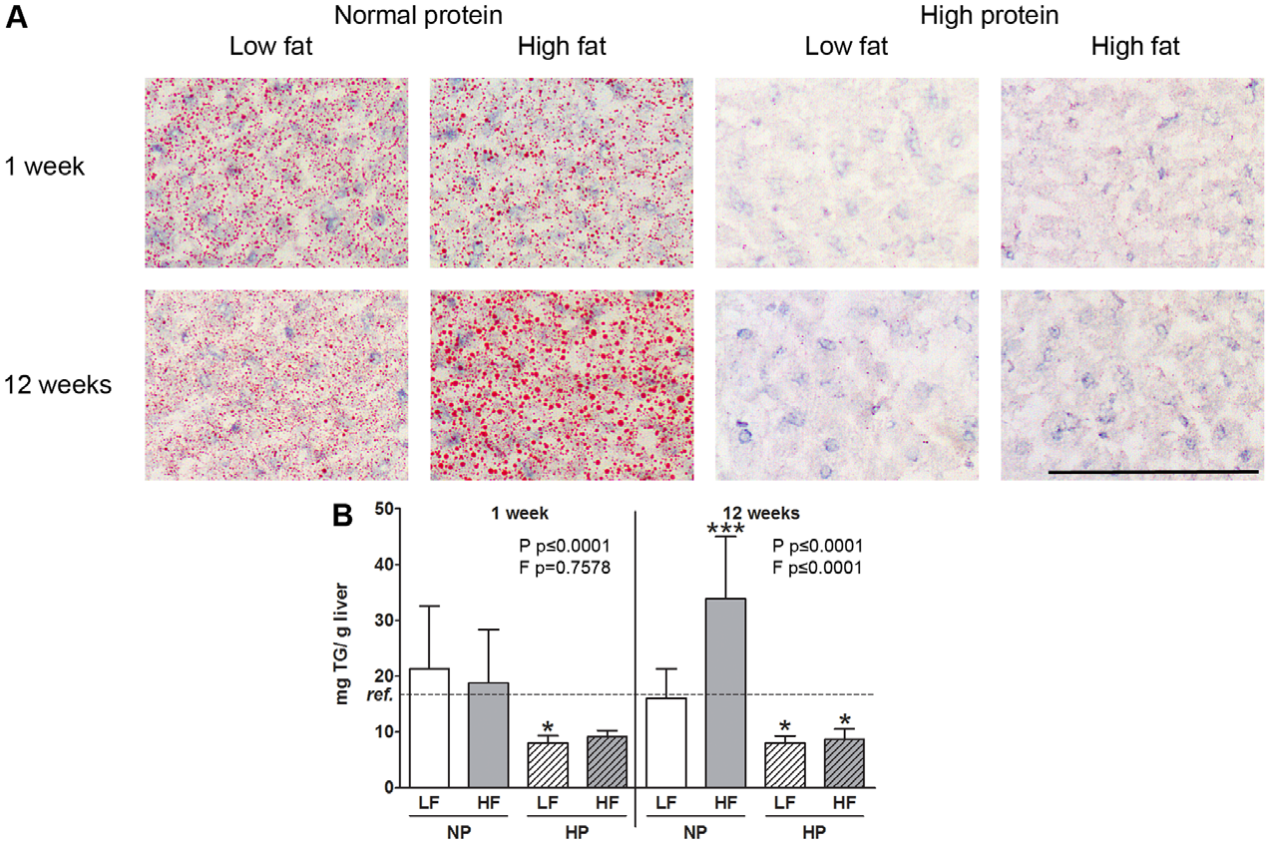
Hepatic steatosis. In mice fed the LF-NP and HF-NP large amounts of lipid droplets can be detected after 1 week and 12 weeks of dietary intervention. Mice fed the high protein diets (LF-HP and HF-HP) these lipid droplets are almost absent. (A) Oil red O staining of representative 5 µm liver sections of the 8 groups. Pictures were taken with a light microscope at 20x magnification. Bar represents 50 µm. (B) Hepatic triglycerides (mean expressed in mg/g liver) are decreased by HP feeding after 1 and 12 weeks and increased by HF feeding after 12 weeks. Effects of increased dietary protein (P) and fat (F) within one time point were tested using general linear model with P and F as fixed factors. * represents values significantly different from the reference group (ref.) as determined by one way ANOVA: *p≤0.05, **p≤0.01, ***p≤0.001.

### Liver phenotyping

Liver dry weight was determined by lyophilisation of samples at −40/−50°C and 10 Torr. Water content was calculated subsequently. Liver protein and triglycerides were determined in 5% liver homogenates prepared in buffer containing 2 mM EDTA and 10 mM Tris at pH 7.5 using enzymatic colorimetric assays (Protein Determination Kit, Cayman Chemical Company, Ann Arbor, Michigan; Triglycerides liquicolor mono, Human Diagnostics, Wiesbaden, Germany). For oil red O/haematoxylin staining frozen livers were sectioned at 5 µM in a Cryostat (Leica Microsystems BV, Rijswijk, the Netherlands), mounted on glass slides and air-dried for 30 minutes at room temperature. Sections were fixed in 4% buffered formaldehyde for 10 minutes prior to staining which was performed using standard protocols. Pictures of liver sections were taken with a light microscope (Olympus BX41, Essex, UK) at 20x magnification.

**Table 2:** Liver weight and composition (means ± SD) of C57BL/6J mice fed four different diets for 1 and 12 weeks.

Liver	Time point	LF-NP	HF-NP	LF-HP	HF-HP	p-value (HP)	p-value (HF)
Total weight (g)	Reference	1.08±0.07	-	-	-	*-*	*-*
	1 week	1.06±0.15	1.07±0.15	1.34±0.07***	1.22±0.14*	≤0.0001***	*0.2339*
	12 weeks	1.11±0.10	1.15±0.12	1.24±0.10*	1.21±0.12	*0.0159* *	*0.7723*
Dry weight (g)	Reference	0.30±0.12	-	-	-	*-*	*-*
	1 week	0.35±0.14	0.39±0.06	0.44±0.06*	0.40±0.05	*0.0866*	*0.9133*
	12 weeks	0.35±0.10	0.40±0.15	0.40±0.10	0.44±0.03*	*0.3963*	*0.3958*
Water content (g)	Reference	0.76±0.13	-	-	-	*-*	*-*
	1 week	0.70±0.16	0.68±0.11	0.90±0.08	0.82±0.10	0.0001***	*0.1662*
	12 weeks	0.76±0.16	0.76±0.13	0.83±0.08	0.78±0.10	*0.0442* *	*0.7989*
Protein content (g/g liver)	Reference	0.79±0.12	-	-	-	*-*	*-*
	1 week	0.76±0.13	0.87±0.12	0.77±0.17	0.83±0.10	*0.7560*	*0.0444* *
	12 weeks	0.84±0.12	0.80±0.10	0.81±0.12	0.85±0.17	*0.8208*	*0.9961*

Groups are compared to the reference group. High protein (HP) and high fat (HF) feeding was tested within one time point.

* expresses significant difference to reference group: *p≤0.05, **p≤0.01, ***p≤0.001.

### VLDL production

VLDL production was determined on an additional set of mice (n = 9/group) after feeding the 4 diets for 1 week. Diets, treatment, food intake and body weight development were highly similar to the first set (data not shown). The experiment started at the beginning of the dark phase. After 12 hours of fasting mice were anaesthetised by an intra-peritoneal injection of hypnorm and dormicum (5x diluted; 0.1 ml/10 g bodyweight). Subsequently the LPL-inhibitor tyloxapol (Triton WR1339, Sigma, 0.5 µg/10 g bodyweight as 15% solution (w/w) in saline) was injected intra-orbitally. Plasma was sampled from tail bleedings and triglyceride and glycerol concentrations were measured every 30 minutes for 180 minutes to determine VLDL production. VLDL-TG production rate was determined from the slope of the curve from all individual mice.

**Figure 3:**
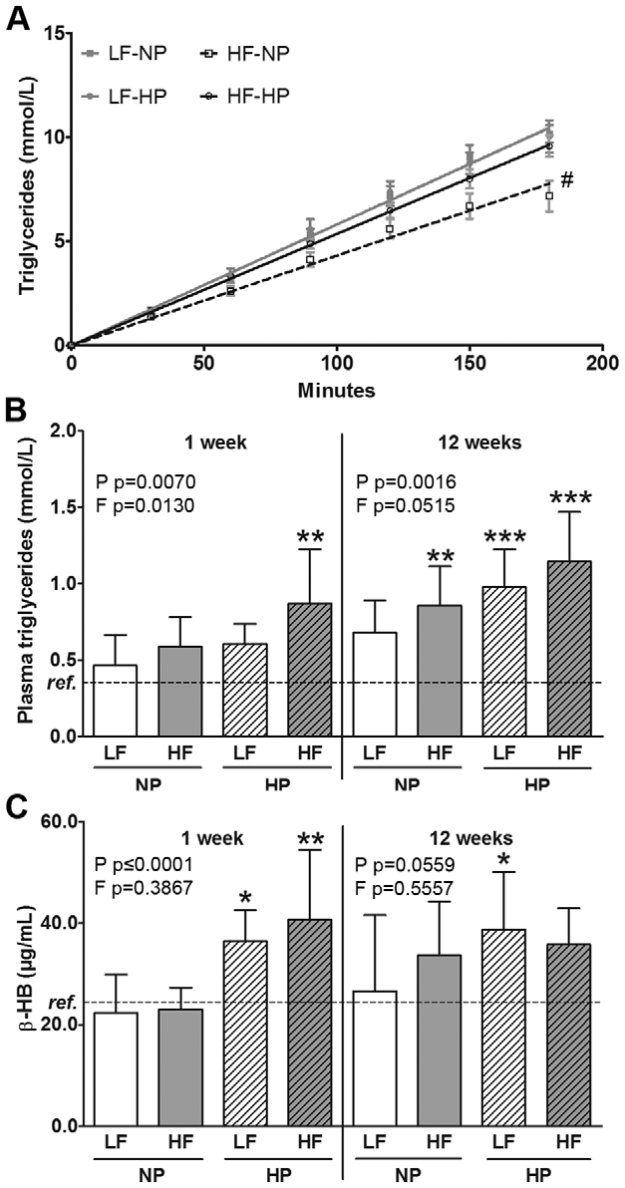
Fasted and postprandial plasma values. Fasted plasma triglyceride concentration after Triton WR1339 injection (A). Hepatic VLDL production rate is lower after 1 week of HF-NP feeding (mean ± SEM). Plasma concentrations 2 hours postprandial to a standardised meal indicating increased TG (B) and BHB (C) in HP-fed mice in comparison with NP-fed mice. Effects of increased dietary protein (P) and fat (F) within one time point were tested using general linear model with P and F as fixed factors. * represents values significantly different from the reference group (ref.) as determined by one way ANOVA: *p≤0.05, **p≤0.01, ***p≤0.001. # represents significant difference from all other groups as determined using one way ANOVA.

### Plasma measurements

Plasma triglyceride concentration was determined using an enzymatic colorimetric test (Triglycerides liquicolor mono, Human Diagnostics, Wiesbaden, Germany). Concentrations were corrected for free plasma glycerol (Free glycerol FS*, DiaSys Diagnostic Systems GmbH, Holzheim, Germany). For determination of plasma β-hydroxybutyrate, the enzymatic assay β-Hydroxybutyrate LiquiColor (Stanbio Laboratory, Boerne, Texas) was used. Plasma leptin was measured using the Leptin (Mouse/Rat) ELISA (ALPCO Diagnostics, Salem, New Hampshire).

**Figure 4:**
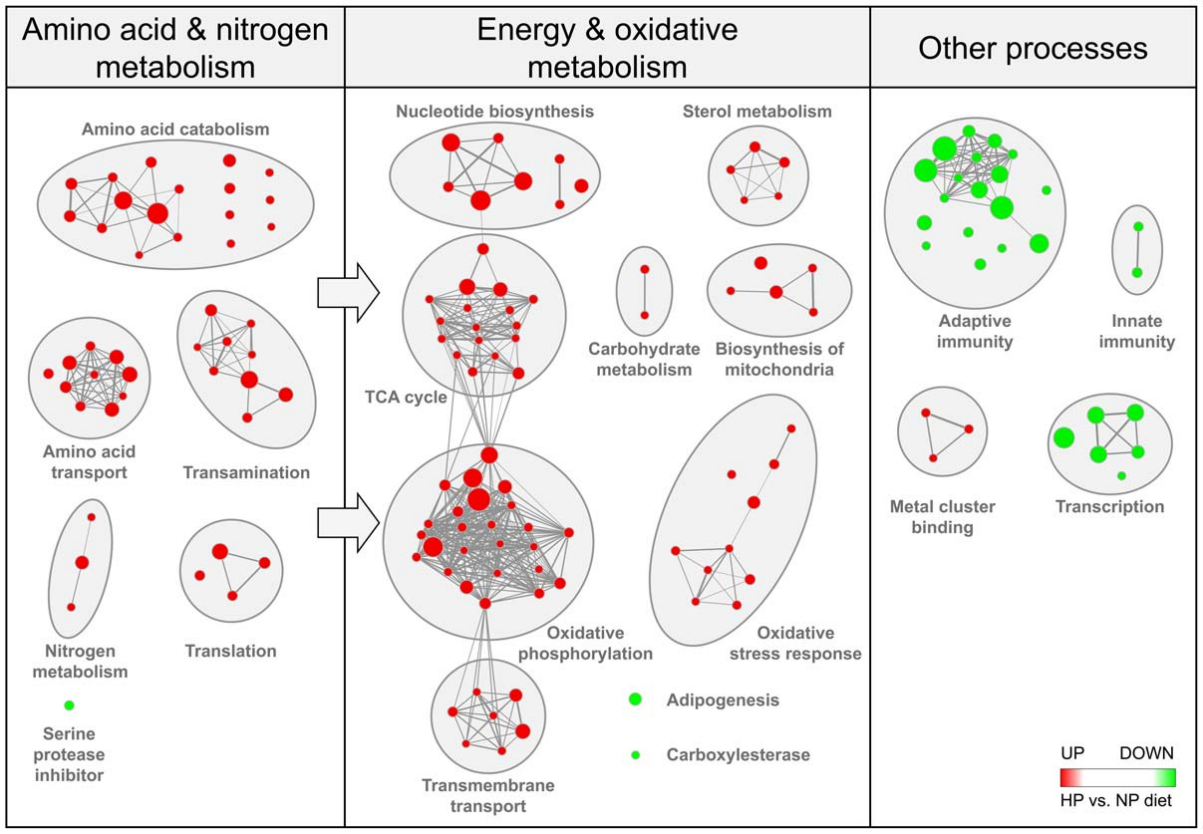
Enrichment map for HP versus NP feeding to identify biological functions. The map displays the enriched gene-sets in HP fed vs. NP fed mice, independent of background diet and time. Nodes represent gene-sets while edges represent overlapping genes. Gene-sets that did not pass the enrichment significance threshold (p≤0.005 and false discovery rate (FDR) ≤0.1) are not shown. Red node colour represents enrichment in HP fed animals (or induction by HP diet), whereas green represents enrichment in NP fed animals (or suppression by HP diet). Clusters of functionally related gene-sets were manually circled and assigned a label.

**Figure 5:**
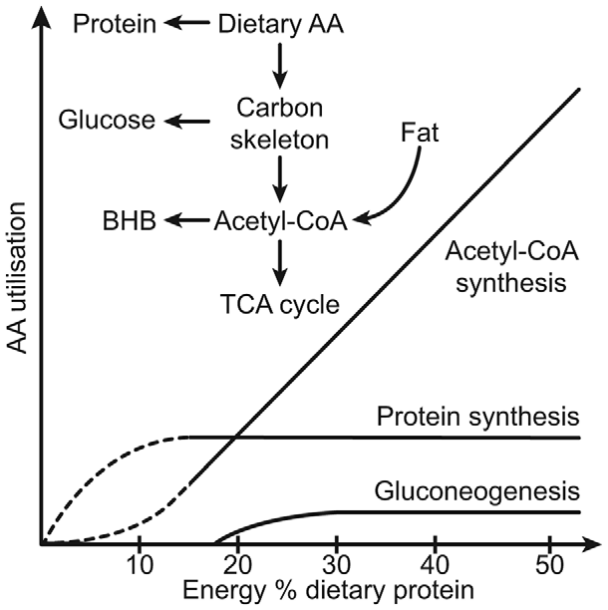
Schematic fate of dietary amino acid utilisation in the liver. Dietary amino acids are firstly used for protein synthesis; however, this can only happen to a limited extent. Subsequently, carbon skeletons can be utilised for gluconeogenesis in a very limited amount. An overload of the liver with dietary amino acids promotes catabolism to acetyl-CoA. Synthesised acetyl-CoA is either channelled into the TCA cycle or used for BHB production. With increasing ingestion of protein, amino acid oxidation and production of BHB from acetyl-CoA becomes more important in relation to gluconeogenesis and protein synthesis.

### Statistical analysis

All data are expressed as means ± SD. Effects of increased dietary protein and fat within one time point were tested for statistical significance using general linear model with HP and HF as fixed factors in PASW Statistics 18.0 software (SPSS Inc., Chicago, Illinois). Mean values were compared to the reference group using one way ANOVA and Dunnett as post hoc test. Body weight was tested using a linear mixed model with diet as main and time as repeated factor. Least significant difference (LSD) test was used as post hoc test. Effects of the diets on food intake and VLDL production rate were tested using one way ANOVA. For all tests, p-values ≤0.05 were considered to be statistically significant.

### Liver transcriptome analysis

Total RNA was extracted from liver tissue with TRIzol reagent (Invitrogen, Breda, the Netherlands) and purified using SV total RNA isolation system (Promega, Leiden, the Netherlands). Concentrations of RNA samples were determined on a NanoDrop ND-1000 UV-Vis spectrophotometer (Isogen, Maarssen, the Netherlands). Total purified liver RNA of 5 mice per group was subsequently pooled at a concentration of 500 ng/mouse. Quality of the pooled RNA was assessed on an Agilent 2100 bioanalyzer with the 6000 Nano Kit using the Eukaryote Total RNA Nano assay (Agilent Technologies, Amsterdam, the Netherlands). RNA was judged as being suitable for array hybridization only if samples showed intact bands corresponding to the 18S and 28S ribosomal RNA subunits, displayed no chromosomal peaks or RNA degradation products and had a RNA integrity number (RIN) above 8.0. The Ambion WT Expression kit (Life Technologies, P/N 4411974) in conjunction with the Affymetrix GeneChip WT Terminal Labelling kit (Affymetrix, Santa Clara, CA; P/N 900671) was used for the preparation of labelled cDNA from 100 ng of total RNA without rRNA reduction. Samples were hybridized on Affymetrix GeneChip Mouse Gene 1.0 ST arrays. Quality control and normalisation were performed using Bioconductor packages integrated in an on-line pipeline [24]. Probe sets were redefined according to Dai et al. [25]. Probes were reorganized based on the Entrez Gene database, build 37, version 2 (remapped CDF v14). Normalised expression estimates of probe sets were computed by the robust multiarray (RMA) analysis algorithm available in the Bioconductor library AffyPLM using default settings [26]. Array data have been submitted to the Gene Expression Omnibus and is available under accession number GSE37897.

### Gene set enrichment analysis (GSEA) and enrichment maps

Changes in gene expression due to dietary protein independent of intervention duration and background diet were related to functional changes using gene set enrichment analysis (GSEA) [27]. For each of the 21 212 genes on the array, the ratio of the mean signal intensity of the HP groups and the mean signal intensity of the NP groups was calculated and ranked. Subsequently signal intensity ratios were analysed for over- or underrepresentation in predefined gene sets derived from Gene Ontology, KEGG, National Cancer Institute, PFAM, Biocarta, Reactome and WikiPathways pathway databases. Only gene sets consisting of more than 15 and fewer than 500 genes were taken into account. The statistical significance of GSEA results was determined using 1 000 permutations. The Enrichment Map plugin for Cytoscape was used for visualization and interpretation of the GSEA results [28], [29]. The enrichment map was generated with gene sets that passed conservative significance thresholds (p-value <0.005, False Discovery Rate (FDR) <0.1, Jaccard Overlap Combined Cut-off >0.375). This resulted in a network of 154 nodes (gene sets), of which 125 were positively and 29 were negatively enriched. Clusters of processes were manually circled and assigned a summarising label. A high resolution version of the enrichment map containing the names of all gene sets can be found in supporting information (Figure S1). For selected clusters of processes, genes contributing to the enrichment of the gene sets were extracted. For each of those genes, fold changes (FC) of signal intensity of the treatment group in comparison with the reference group were calculated and expressed in a heat map (Table S1). Additionally, genes contained in gene sets describing fatty acid oxidation are expressed in table S1.

## Results

### HP feeding reduced high fat diet-induced body weight gain

Cumulative food intake did not differ between the dietary groups (p = 0.3235) (Figure 1 A). However, only mice fed the HF-NP, but not those fed the HF-HP diet, gained significantly more weight (p<0.0001) compared to the other groups during the entire dietary intervention period (Figure 1 B). This was accompanied by a high fat-induced larger epididymal adipose tissue mass after 12 weeks (p = 0.0003, Figure 1 C). After increasing dietary protein content, adipose tissue mass of HP-fed mice was significantly lower compared to NP-fed mice already after 1 week of diet (p = 0.0001). This effect was even stronger after 12 weeks (p≤0.0001). In line with these findings plasma leptin concentrations were significantly elevated by high fat feeding (p = 0.0108) over 12 weeks, while they were significantly lower after high protein feeding (p≤0.0001, Figure 1 D).

### HP feeding induced lower intrahepatic lipid accumulation

Augmenting dietary protein leads to an increase in liver weight compared to the reference group after both 1 and 12 weeks of diet (Table 2). The higher liver weight might partly be explained by increased water content in response to a HP diet after 1 week (p = 0.0001) and 12 weeks (p = 0.0442). Effects on dry weight are less pronounced although a significant increase after 1 week in the LF-HP group and after 12 weeks was detected in the HF-HP group compared to the reference group. Liver protein content was increased by HF feeding only after 1 week (p = 0.0444). To investigate the effect of high protein feeding on hepatic steatosis, frozen liver sections were stained for lipids with oil red O. Mice fed the LF-NP and HF-NP diet show signs of hepatic steatosis characterised by lipid droplets after 1 week of the dietary intervention and maintained it for the following weeks (Figure 2 A). Animals fed the high protein diets (LF-HP and HF-HP) were visibly protected from hepatic lipid accumulation as shown by the absence of lipid droplets on the liver sections. Quantitaive measurements demonstrated that hepatic triglyceride levels were only significantly elevated in the HF-NP group after 12 weeks intervention compared to the reference group (Figure 2 B). At the same time HP feeding significantly lowered hepatic triglycerides both after 1 and 12 weeks (p≤0.0001) independent of the fat content of the background diet.

### HP feeding restored the HF-induced reduction in VLDL production rate and increased postprandial plasma TG and β-hydroxybutyrate

Hepatic VLDL production was determined in fasted mice after 1 week of dietary intervention (Figure 3 A). VLDL production rate of HF-NP fed mice was decreased by 26% compared to LF-NP animals (2.59±0.09 vs. 3.49±0.07 mM/h for HF-NP and LF-NP, respectively, p = 0.0234). This effect was blunted when fed a HF-HP diet. Also in the postprandial state, after 1 and 12 weeks of the respective diets, TG levels were significantly elevated by HP feeding (p = 0.0070 and p = 0.0016, respectively, Figure 3 B). HF feeding had only a significant effect on increased plasma TG after 1 week (p = 0.0130). β-hydroxybutyrate (BHB) significantly increased in response to HP feeding after 1 week of diet (p≤0.0001, Figure 3 C). After 12 weeks the protein effect was not significant anymore (p = 0.0559). However, compared to the reference group, animals fed the LF-HP and HF-HP diet for 12 weeks had around 50% higher plasma BHB levels (p = 0.0090 and p = 0.0557, respectively).

### Hepatic gene expression in response to HP feeding

Hepatic gene expression was determined by microarray analysis of pooled RNA samples. Gene set enrichment analysis revealed several clusters of processes being enriched as a result of HP feeding independent of the background diet and the time point (Figure 4). A first cluster of processes enriched in the liver of HP-fed animals compared to NP fed animals is involved in amino acid and nitrogen metabolism (amino acid catabolism, amino acid transport, transamination, nitrogen metabolism, translation and serine protease inhibitor). Gene sets describing energy and oxidative metabolism (nucleotide biosynthesis, TCA cycle and oxidative phosphorylation, biosynthesis of mitochondria, oxidative stress response, sterol metabolism) were likewise enriched under HP-feeding while adipogenesis and carboxylesterase were enriched under NP conditions. Other gene sets enriched by NP feeding compared to HP feeding include those related to immune function such as adaptive and innate immune response and activity of transcription factors as well as induction of genes related to metal cluster binding. Details for all processes as well as heat maps of genes responsible for the enrichment of selected clusters can be found under supporting information (enrichment map with gene set names, Figure S1; heat maps of selected gene sets, Table S1). Gene sets describing *de novo* lipogenesis and fatty acid oxidation were not significantly enriched. All genes contained in gene sets describing fatty acid oxidation are expressed in table S1.

## Discussion

This study confirms that mice fed a high fat-normal protein diet gained significantly more weight, adipose tissue mass and liver fat compared to all other groups despite a similar energy intake [16], [17]. It further shows that in contrast to fat a high protein content in the diet induced a lower weight gain, a lower adipose tissue mass and a lower accumulation and storage of fat in the liver compared to all other groups. Interestingly the present study shows that the reducing effect of protein on adipose tissue mass and liver fat occurred both under high and low fat conditions. This suggests a specific effect of protein in liver independent of the fat and carbohydrate content of the diet. These results are in line with previous observations in which dietary protein reduced adiposity in rats [23], [30] and humans [31], [32] during short term interventions. Increasing protein intake was also reported to reduce liver fat accumulation in rats [23] and mice [22] as well as in the context of high fat diet in human [15]. This was earlier interpreted as both a direct and indirect action of AA. However, the question why energy in the form of amino acids is less efficiently transferred to fat in the liver still remained unsolved [33].

The present results are in agreement with the idea that in order to handle the overflow of incoming AA under HP feeding, AA uptake capacity in the liver increases. Also hepatic genes involved in AA deamination and metabolism are induced whereas the use of AA for liver protein synthesis mainly remains unaffected. The results of GSEA revealed that HP feeding both in context of a high fat or a high carbohydrate diet strongly affects hepatic gene expression in mice. This effect includes induction of gene sets belonging to AA processing pathways such as AA transport and catabolism, transamination processes and nitrogen metabolism. In previous observations HP diet enhanced liver transporter activity and AA uptake in rats [30]. Accordingly, the present study shows that liver expression of membrane transport proteins of the solute carrier (Slc) family was enhanced in mice adapted to the HP diet. It was also earlier observed in rats that the increased quantity of AA taken up by the liver was unable to further increase liver protein synthesis compared to a normal protein diet. The excess of AA was thus not used for protein synthesis but subjected to deamination with the release of amino acid-derived carbon skeleton [34], [35], [36]. Extending these observations, the present study shows no modification of genes involved in liver protein synthetic pathways, whereas there is an induction of catabolic gene sets (Figure 4). They contain a large number of amino transferases and transaminases (Table S1, AA_catabolism) as well as gene sets involved in metabolism of single AA, amongst others histidine, glycine, arginine and phenylalanine.

In contrast to earlier findings, in the present study GSEA did not reveal any effects of the HP diet on *de novo* lipogenesis [37], [38]. In our analysis we focused on the effect of the high protein diet independent from the background diet. *De novo* lipogenesis was repeatedly demonstrated to be reduced during high fat feeding [39], [40], [41] and is possibly a result of decreased carbohydrate intake. This effect should therefor occur in both HF groups and the possible additional effect of increasing dietary protein plays a less important role under these conditions.

It was also previously reported in rats that in response to HP feeding hepatic gluconeogenesis remains induced in the fed state [42]. Our observations in mice reveal an induction of gene sets affecting carbohydrate metabolism and more specifically genes linked to gluconeogenesis (Figure 4). However, it also has been reported that the amount of AA-derived carbon skeletons used for gluconeogenesis is very limited and this pathway cannot cope with the excess of incoming AA under HP feeding [43]. The present study indicates that in order to handle the excess of AA taken up by the liver that are not used for protein synthesis or gluconeogenesis under HP feeding, hepatic capacity for AA oxidative deamination processes increases (Figure 4). Indeed, biosynthesis of mitochondria and sets of genes encoding enzymes of the TCA cycle and oxidative phosphorylation were induced in the liver by a HP diet in mice. Via transamination reactions AA are metabolised into α-keto acids, then converted into acetyl-CoA, succinyl-CoA, fumarate, α-keto glutarate and oxaloacetate which subsequently enter the mitochondrial TCA cycle. The direct supply of dietary AA into the TCA cycle when fed a HP diet was described earlier [43]. NADH is generated in the TCA cycle, which is a precursor required for oxidative phosphorylation and generation of ATP (Table S1, TCA_cycle). Genes related to oxidative phosphorylation are encoding different sub-complexes of enzymes like cytochrome c oxidase, NADH dehydrogenase and ATP synthase (Table S1, oxidative_phosphorylation). An energy restricted HP diet was previously described to induce a stronger increase in mitochondrial oxidation compared to a normal energy restricted diet [44]. Moreover, in rats adapting to a HP diet a transient drop in carbohydrate oxidation and increase in protein oxidation was observed [42], [43]. In humans, both without and after adaptation to a HP diet, basal protein oxidation and post-prandial energy expenditure in response to a HP meal were higher compared to the control condition [45]. Fatty acid oxidation was not detected to be altered in the present study. This is in line with previous data from rats which demonstrated that postprandial lipid oxidation increases on the first day of a HP diet, but returns to baseline during adaptation [42]. In human post prandial lipid oxidation was even suggested to decrease after adaptation to a HP diet [45]. Tremendous increases in acetyl-CoA, inhibiting β-oxidation may explain these findings.

Interestingly, the HP diet increased plasma β-hydroxybutyrate (BHB) levels, suggesting the induction of a fasting-like situation and a channelling of the excess of acetyl-CoA to BHB synthesis. It is likely that during HP feeding the increased catabolism of AA acids induces an overflow of acetyl-CoA exceeding the cellular energy requirements. In the liver it is not converted into fat but into BHB that is excreted and may act as energy source for extrahepatic organs. The HP diet increased postprandial plasma TG which suggests a larger excretion of hepatic lipids via VLDL which at the same time decreases lipid storage. Indeed VLDL production rate decreases with a HF-NP diet but this effect is restored under HF-HP conditions. In humans increased postprandial TG levels in response to a HP adaptation were likewise observed [45]. Although an increased formation of apolipoproteins is not detected on a transcriptional level, this does not exclude the possibility that due to the high influx amino acids lipoprotein synthesis will increase. This will stimulate hepatic formation and excretion of VLDL particles. Other processes enriched under HP conditions are related to oxidative stress response. The high rate of mitochondrial oxidation confronts the liver with large amount of reactive electrophiles and changes the redox state of hepatocytes leading to an activation of Nrf2 [46]. Part of the Nrf2 target genes belong to the glutathione (GSH)-dependent detoxification system [47], encompassing amongst others glutathione synthase and glutathione peroxidases for neutralising oxidative stress (Table S1, oxidative_stress_response). Moreover, several genes of the glutathione S-transferase (GST) family are overexpressed under HP feeding. GST catalyses the conjugation of reduced GSH to electrophiles, e.g. lipid peroxides, thereby protecting against peroxidation [48]. GSH is regenerated by glutathione reductase (GSR). The hepatic synthesis of GSH uses AA (cysteine, glutamate and glycine) provided by the HP diet [48]. These processes require energy in form of ATP, which is not available for other metabolic functions or storage as lipid.

Taken together, the present data suggest that at a similar energy intake, mice ingesting HP as compared to NP diets possess a lower body weight, adiposity and hepatic fat due to a combination of modulated processes (Figure 5). Firstly, the hepatic capacity to store AA as protein is limited and the excess AA is deaminated possibly to prevent a rise in cellular and blood AA concentration. Secondly, as the transfer of AA-derived carbon to glucose also is limited and *de novo* lipogenesis seems absent, these carbon skeletons are metabolized and transferred to acetyl-CoA. Thirdly, as result of an overload of acetyl-CoA and limited capacities to further metabolise it, energy is excreted from the liver in form of BHB and can act as a substrate to extrahepatic organs. In addition, more energy has to be used for detoxification of electrophiles produced during protein catabolism. Thus, handling of large amounts of amino acids leads to an increased energy utilisation in the liver. At the same time hepatic lipid storage is reduced. Additional effects contributing to the leaner phenotype of HP fed mice might be a slight, non-significant reduction in food intake, modifications in intestinal microbiota resulting in reduced nutrient availability as well as increased thermogenesis and therefore easier maintenance of body temperature at 20°C. This, however, has to be subject to further investigation. At last, HP in comparison with NP feeding promoted down regulation of both adaptive and innate immunity. Fatty liver is often associated with inflammation [16]. The diminished hepatic lipid content in HP-fed animals prevented an increase in inflammation markers and a response of the immune system, even explicit inflammation was not yet detected in NP fed mice.

In conclusion, the liver plays a buffering role in preventing the extrahepatic tissues from an overload of AA. Moreover, a shift in energy from the liver to peripheral tissues and its use for example in muscle are suggested and should be further investigated also on a longer term.

## Supporting Information

Figure S1 **Enrichment map for HP versus NP feeding to identify biological functions.** The map displays the enriched gene-sets and their labels in HP fed vs. NP fed mice, independent of background diet and time. Nodes represent gene-sets while edges represent overlapping genes. Gene-sets that did not pass the enrichment significance threshold (p≤0.005 and false discovery rate (FDR) ≤0.1) are not shown. Red node colour represents enrichment in HP fed animals (or induction by HP diet), whereas green represents enrichment in NP fed animals (or suppression by HP diet). Clusters of functionally related gene-sets were manually circled and assigned a label.
(PDF)

Table S1 **Heat maps of genes contributing to the enrichment of the gene sets within selected clusters of processes.** For each of those genes, fold changes (FC) of signal intensity of the treatment group in comparison with the reference group at week 0 were calculated and expressed in a heat map. Red colour represents up regulation and green colour represents down regulation of a gene in comparison with the reference group. Additionally, genes contained in gene sets describing fatty acid oxidation are expressed.
(XLSX)
