# Supplementary material for: Dietary Protein Affects Gene Expression and Prevents Lipid Accumulation in the Liver in Mice
Source: PLoS One. 2012 Oct 23;7(10):e47303. doi: 10.1371/journal.pone.0047303 (PMC3479095; doi:10.1371/journal.pone.0047303)
Supplement: Figure S1 — Enrichment map for HP versus NP feeding to identify biological functions. The map displays the enriched gene-sets and their labels in HP fed vs. NP fed mice, independent of background diet and time. Nodes represent gene-sets while edges represent overlapping genes. Gene-sets that did not pass the enrichment significance threshold (p≤0.005 and false discovery rate (FDR) ≤0.1) are not shown. Red node colour represents enrichment in HP fed animals (or induction by HP diet), whereas green represents enrichment in NP fed animals (or suppression by HP diet). Clusters of functionally related gene-sets were manually circled and assigned a label. (PDF) [file pone.0047303.s001.pdf]

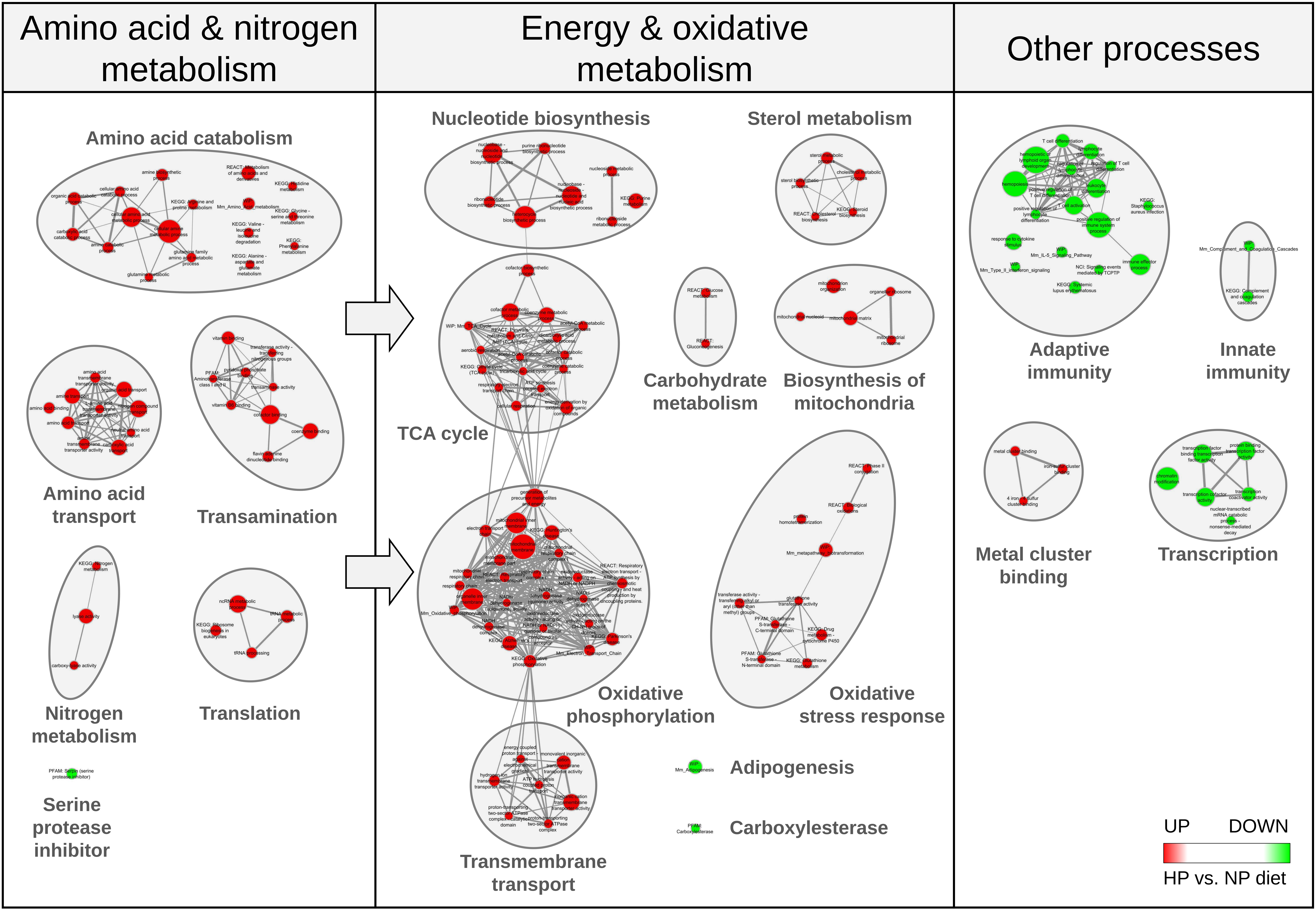

Amino acid & nitrogen metabolism
Energy & oxidative metabolism
Other processes
Amino acid catabolism
Amino acid transport
Transamination
Nitrogen metabolism
Translation
Serine protease inhibitor
Nucleotide biosynthesis
Sterol metabolism
TCA cycle
Carbohydrate metabolism
Biosynthesis of mitochondria
Oxidative phosphorylation
Oxidative stress response
Transmembrane transport
Adipogenesis
Carboxylesterase
Adaptive immunity
Innate immunity
Metal cluster binding
Transcription
UP
DOWN
HP vs. NP diet
